# Resilience Shields and Grit Intensifies Subjective Cognitive Decline in Middle‐To‐Older Aged Black Americans

**DOI:** 10.1002/gps.70214

**Published:** 2026-04-18

**Authors:** Sunkanmi Folorunsho, Maria Clark, Obinna Odo, Yolanda L. Jackson, Candidus Nwakasi, Darlingtina K. Esiaka

**Affiliations:** ^1^ Department of Sociology University of Nebraska–Lincoln Lincoln Nebraska USA; ^2^ Department of Behavioral Science University of Kentucky College of Medicine Lexington Kentucky USA; ^3^ Center for Health, Engagement, and Transformation University of Kentucky College of Medicine Lexington Kentucky USA; ^4^ Department of Sociology and Gerontology Miami University Oxford Ohio USA; ^5^ Scripps Gerontology Center Miami University Oxford Ohio USA; ^6^ College of Communication and Information University of Kentucky Lexington Kentucky USA; ^7^ Center for Clinical and Translational Science University of Kentucky College of Medicine Lexington Kentucky USA; ^8^ Department of Human Development and Family Science University of Connecticut Storrs Connecticut USA; ^9^ Sanders‐Brown Center on Aging University of Kentucky Lexington Kentucky USA

**Keywords:** black adults, black adults'brain health, cognitive decline risk, psychosocial factors, resilience in black adults

## Abstract

**Background:**

There is growing interest on the relationship between psychosocial factors and cognitive health trajectories in adulthood. While grit and resilience have been shown to influence health outcomes, their differential effects on subjective cognitive decline (SCD) in Black American adults remain understudied. The purpose of this study is to help fill this gap.

**Methods:**

The study utilized a cross‐sectional research design. Black American participants (*N* = 242; Mean age = 44.32) responded to a survey assessing SCD, grit, resilience, and sociodemographic factors. Linear regression analysis was performed to test the associations between grit and resilience and SCD, and to explore if the associations differed by sex.

**Results:**

Analysis showed that grit (*β* = 0.386, *p* < 0.001) was positively associated with SCD, but the effect size was larger in Black men (*β* = 0.527, *p* < 0.001) than women (*β* = 0.285, *p* < 0.001). Also, resilience (*β* = −0.220, *p* < 0.001) was negatively associated with SCD and the effect size was larger in Black men (*β* = −0.266, *p* < 0.001) than women (*β* = −0.187, *p* < 0.01).

**Conclusion:**

Our findings suggest that while higher levels of resilience may be a protective factor against SCD, higher grit may be associated with greater likelihood of SCD. Future studies should assess whether these associations persist when using objective measures of cognitive decline and further examine these factors as potential targets for cognitive health interventions.

## Key Points

- Higher resilience is associated with lower subjective cognitive decline among Black American adults.
- Higher grit correlates with increased perceived cognitive decline, with a stronger association in Black men.
- Building psychological resilience is a promising target for culturally tailored brain‐health interventions.

## 1 Introduction

Subjective cognitive decline (SCD), a self‐reported worsening in memory or thinking ability, has gained attention as a potential early marker of neurodegenerative cognitive disease before clinical impairment is evident [1, 2]. Research shows that individuals with persistent SCD are at elevated risk of future cognitive decline and dementia. Specifically, people with sustained SCD had over a 75% probability of developing mild cognitive impairment (MCI) or dementia within a decade​ and 82% of people with SCD progressed to dementia over 12 years [2]. This makes SCD one of the reliable predictors of eventual dementia, even more so than certain objective measures such as neuropsychological test performance and hippocampal atrophy on magnetic resonance imaging [2]. Early identification of SCD is especially relevant for Black American adults, who face a disproportionate burden of cognitive impairment, including MCIs and dementia compared to non‐Hispanic White Americans [2, 3]. While evidence on SCD in Black adults is limited [4], studies have attributed the disparities in cognitive impairment evident among Black American adults to be influenced by, but not limited to genetics [5], immunological factors [5], lifestyle and behavior [6], exposure to stressors [7] and psychosocial factors [9].

There is growing research interest on the relationship between psychosocial factors and cognitive health trajectories in adulthood [8]. In particular, grit and resilience are thought to help individuals cope with stressors and potentially buffer against cognitive decline [9]. *Grit* is defined as perseverance and passion for long‐term goals and reflects a tendency to maintain effort and interest over years despite setbacks [10]. Resilience refers to the ability to “bounce back” or recover after stress and adversity [11]. Grit and resilience encompass adaptive coping that allows individuals to maintain or regain psychological well‐being in the face of hardships. Although grit and resilience are conceptually distinct, their close theoretical and empirical overlap warrants examining them jointly, as complementary constructs within the same analytic framework, rather than treating one solely as a confounder of the other.

High resilience may serve as protective factors that promote cognitive health by helping individuals manage stress, engage in healthy behaviors, or persevere in mentally stimulating activities. For instance, studies have found that greater psychological resilience was associated with better global cognitive function [12] and that resilience during midlife may delay or prevent cognitive decline in later years [13]. However, a recent study that examined active coping strategies (i.e., deliberate actions taken to manage stress and difficult situations such as seeking support or reframing the situation in a positive light) as a form of resilience found that while higher active coping may reduce the likelihood of SCD, higher levels of grit were associated with greater SCD [14, 15]. Although grit and resilience share a common emphasis on persistence, they are conceptually distinct: grit reflects a stable trait‐level disposition toward sustained effort and passion for long‐term goals regardless of context, whereas resilience centers on adaptive recovery and continued functioning following disruption. [10, 11]. This distinction is important because trait‐level perseverance (grit), particularly under conditions of chronic stress, may not confer the same adaptive benefits as situational resilience, and may instead amplify exposure to stress‐related cognitive burden.

Psychosocial factors may play a particularly crucial role in the cognitive health of Black American adults, who often face significant life stressors and rely on strong coping mechanisms and support systems to foster resilience [16]. However, exposure to cumulative adversity such as socioeconomic challenges and racism‐related stress [17, 18, 19] and high‐effort coping (e.g., persistently striving to overcome barriers despite chronic stress or working significantly harder than peers to counteract systemic barriers) are known to negatively impact health including cognitive functioning [20]. In a cohort of older Black adults, relentlessly persevering under great stress was associated with lower baseline cognitive function [20].

While the effect of grit and resilience on cognitive function have been explored in aging research, there remains a critical gap in understanding their effect among middle‐to‐older aged Black Americans, particularly in the context of SCD. This is important in Black American populations where cultural and social influences may shape how people apply different stress coping strategies, perceive and report cognitive concerns [21]. Thus, this study examined the effects of grit and resilience on SCD in middle to older age Black adults, as this age range captures individuals in midlife and beyond when early signs of cognitive change may emerge. Also, Black Americans are at higher risk of developing chronic conditions (e.g., hypertension, diabetes, cardiovascular disease, and obesity) that are established precursors to cognitive decline during middle age and experience disproportionate levels of early‐onset of cognitive decline, making middle age a critical period for examining potential protective and risk factors.

## 2 Methods

### 2.1 Participants and Procedure

We collected data from participants recruited via Connect, a panel service within the CloudResearch platform that links researchers with community‐dwelling adults interested in academic studies [22]. For this study, we created a project listing on Connect, which was visible to eligible panel members who could voluntarily sign up to participate. We provided a secure Qualtrics link to the survey directly on the platform, and survey participation was limited to individuals who met the study's inclusion criteria as determined by CloudResearch's prescreening system. Participants were specifically recruited based on the following eligibility criteria: self‐identify as Black American adults and being able to read and understand English at a level necessary to provide appropriate consent for research participation.

Participant IP addresses were verified, to ensure U.S. based participation and an IP address blocker was used to avoid repeated entries from the same individuals. Sporadic in‐survey attention checks were also utilized, and participants received monetary compensation for survey completion. The three measures reported in this current study are part of a larger battery of measures (examining social and psychological determinants of brain health in Black communities) that took participants an average of 90 min to complete. This study was approved by the Institutional Review Board of Rutgers University (#Pro2023000904).

### 2.2 Measures

#### 2.2.1 Subjective Cognitive Decline (SCD)

This was measured using a 21‐item self‐report questionnaire [23] used to assess memory changes, everyday cognition, and memory functioning. Fifteen of the questions have dichotomous responses (yes = 1/no = 0) and aim to identify if a particular concern has ever been self‐observed. Sample questions are: “Do you have complaints about your memory in the last 2 years?” “Do you feel you are forgetting where things were placed?” The remaining six questions are measured on a Likert scale (i.e., always = 2, sometimes = 1, or never a problem = 0). Samples of these scaled questions are: “How often is the following a problem for you: Knowing whether you've already told someone something?” and “How often is the following a problem for you: Phone numbers you use frequently?” SCD score was computed for each individual by summing their score on the questions, with higher scores indicating a worse SCD (range = 0–27). The mean score for this sample was 6.82 (SD = 5.84). The scale yielded a reliability score of Cronbach's *α* = 0.90.

#### 2.2.2 Grit

Operationalized as the capacity to persevere toward long‐term goals and was evaluated using the Short Grit Scale (Grit‐ S) – and eight item self‐reported assessment [2]. The Grit‐S measures the self‐reflective tendency for persistence toward long‐term goal through two dimensions: consistency of interests (e.g., “I often set a goal but later choose to pursue a different one.”) and perseverance of effort (e.g., “I am a hard worker.”). Four items on the scale were reverse coded. All items were measured on a five‐point Likert scale ranging from (1–not like me at all to 5–very much like me). The scale yielded a reliability score of Cronbach's *α* = 0.85. The combined scores for the dimensions range from 10 to 40, with higher scores indicative of higher grit levels. The mean score for this sample was 26.16 (SD = 3.83).

#### 2.2.3 Resilience

Operationalized as the ability to positively adapt and recover from adverse events. This was assessed using the Connor‐Davidson Resilience Scale (CD‐RISC‐10) [24], an adapted 10‐item scale from the original 25 item CD‐RISC full scale. All 10 items are self‐reported items that are scored on a 5‐point Likert scale (0‐Not true at all, 1‐rarely true, 2‐sometimes true, 3‐often true, and 4‐true nearly all the time). Questions are geared toward participant's self‐reflection and include items: “Able to adapt to change” and “Tend to bounce back after illness or hardship.” The total score of the scale ranged from 0 to 40, with a higher score indicating greater resilience. The mean score for this sample was 28.13 (SD = 7.66). The scale yielded a reliability score of Cronbach's *α* = 0.92.

#### 2.2.4 Demographic Characteristics

Four demographic variables were included in the analyses: sex, age, marital status, and subjective socioeconomic status. Subjective socioeconomic status was assessed by asking participants to rate their socio economic standing on a 10‐rung ladder relative to others in their communities. Age and subjective socioeconomic status were included as continuous variables. Sex and Marital status (single/divorced/widow/separated = 0, married/living as married = 1) were included as dichotomous variables. These covariates were selected based on their established associations with both psychosocial functioning and cognitive aging outcomes in prior literature. Age was included because cognitive concerns increase with advancing age [2]. Education was included as a proxy for cognitive reserve, which is known to moderate the onset and progression of cognitive decline [25]. Marital status was included given evidence that social partnership buffers against cognitive decline, potentially through social engagement and support [26]. Subjective socioeconomic status was included to capture perceived material hardship, a key structural determinant of cognitive health disparities in Black populations [19]. Additionally, two memory self‐appraisal items — an overall rating of current memory functioning and a rating of memory compared to 1 year prior — were included as covariates to statistically control for baseline subjective memory perceptions that might otherwise confound the associations between grit, resilience, and SCD.

### 2.3 Data Analysis

Summations of participant's scores were calculated for each individual assessment. We performed descriptive analysis to identify and categorize our sample by demographic and social characteristics. Linear regression analysis was performed to test the associations between resilience, grit, and SCD in all participants. As an exploratory analysis, we also stratified by sex. Additionally, we performed an Independent‐sample *t*‐Test to examine whether there are sex differences in SCD, grit and resilience. We found no statistically significant differences and thus, did not report the results of the *t*‐test in the result section. To evaluate whether the associations between grit, resilience, and SCD differed significantly by sex, we tested interaction terms (grit × sex; resilience × sex) in the overall model; sex‐stratified analyses were conducted as an exploratory step to further characterize any observed differential effects. We determined statistical significance with the probability of a Type I error set at *p* ≤ 0.05 and conducted all statistical analyses using SPSS version 30.0 (SPSS Inc., Chicago, IL).

## 3 Results

### 3.1 Demographic and Social Characteristics

The distribution of the demographic and social characteristics for the total sample is shown in Table 1. The study included a sample (*N* = 242) of Black men (*n* = 95) and women (*n* = 147) with a mean age of 44.32 (SD = 8.60) years, ranging from 27 to 79 years. Approximately one‐third of the sample (33.1%) were under 40 years of age, 52.2% were between 40 and 54 years old, and 13.5% were aged 55 years or older, with 5.7% aged 60 or above. Most of the sample (88.4%) reported having more than 12 years of education. Of the total sample, 50.8% reported their marital status as single, divorced, or widowed. The average score for subjective social standing rating was 5.02.

**TABLE 1:** Descriptive result of the demographic and social characteristics of participants in the study (*N* = 242).

	Overall (*N* = 242)		Men (*n* = 95)		Women (*n* = 147)		
Variables	Mean (*n*)	SD (%)	Mean (*n*)	SD (%)		Mean (*n*)	SD (%)
Age	44.32	8.60	43.63	8.72		44.76	8.53
Education > 12 years	(214)	(88.4)	(81)	(85.2)		(133)	(90.5)
Marital status (single, divorced or widowed)	(123)	(50.8)	(54)	(56.8)		(65)	(44.2)
SSS (range 0 – 10)	5.02	1.89	5.28	2.00		4.84	1.79
SCD (range 0 – 27)	6.79	5.84	6.39	5.96		7.05	5.77
Grit (range 10 – 40)	26.16	3.83	26.28	4.37		26.08	3.46
Resilience (range 0 – 40)	28.13	7.67	28.79	7.46		27.71	7.79

Abbreviations: *n* = number. SCD = subjective cognitive decline. SD = standard deviation. SSS=Subjective socioeconomic standing.

### 3.2 Associations Between Grit, Resilience, and SCD

**FIGURE 1:**
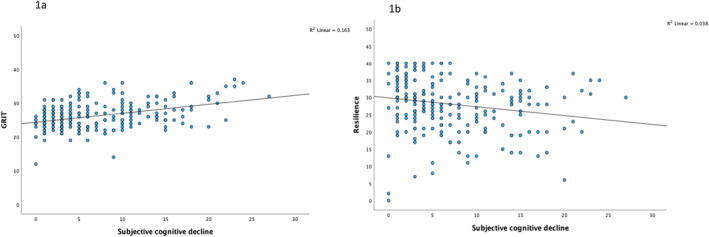
Association between Grit (1a) and Resilience (1b) and Subjective cognitive decline in study sample.

After controlling for demographic factors, we found that higher levels of grit (Figure 1a) are positively associated with higher SCD scores (*β* = 0.386, *p* = < 0.001), with higher scores reflecting greater perception of cognitive decline. This relationship was stronger in men (*β* = 0.527, *p* = < 0.001) than in women (*β* = 0.285, *p* = < 0.001). Conversely, resilience showed an opposite trend (Figure 1b), with increased resilience negatively associated with SCD scores (*β* = −0.220, *p* = < 0.001), therefore, lesser perception of cognitive decline. This relationship was stronger in Black men (*β* = −0.266, *p* = < 0.002) than in women (*β* = −0.187, *p* = < 0.003). See Table 2 for additional information.

**TABLE 2:** Linear regression of association of subjective cognitive decline with grit and resilience stratified by sex.

	Overall (*n* = 242)			Men (*n* = 97)			Women (*n* = 147)		
Variables	*β*	SE	*p*	*β*	SE	*p*	*β*	SE	*p*
Age	−0.050	0.031	0.274	0.026	0.053	0.739	−0.108	0.039	0.065
Education	0.018	0.115	0.697	0.062	0.200	0.471	−0.015	0.147	0.793
Marital status	0.021	0.611	0.687	0.067	1.12	0.475	−0.033	0.754	0.611
SES	0.182	0.184	0.002	0.216	0.316	0.045	0.155	0.241	0.041
Overall memory rating	−0.547	0.829	< 0.001	−0.332	1.99	< 0.001	−0.640	0.945	< 0.001
Memory rating compared to 1 year	−0.098	0.438	0.053	−0.158	0.755	0.105	−0.075	0.611	0.239
Grit	0.386	0.072	< 0.001	0.527	0.114	< 0.001	0.285	0.096	< 0.001
Resilience	−0.220	0.036	< 0.001	−0.266	0.062	< 0.001	−0.187	0.045	0.003

Abbreviations: *β* = Standardized beta. SE = standard error. SES = subjective socioeconomic standing. “Overall memory rating” refers to participants' self‐rated current memory functioning; “Memory rating compared to 1 year” refers to participants' subjective assessment of how their memory compares to the prior year (both items from the SCD questionnaire).

## 4 Discussion

The current study examined the effects of grit and resilience on subjective cognitive decline (SCD), an early indicator of cognitive impairment and dementia, in middle to older age Black Americans. We found that higher levels of grit are associated with higher SCD scores, while higher level of resilience was associated with lower SCD.

Our finding that increased resilience was associated with higher perceived cognitive functioning aligns with previous literature on the association between resilience and subjective cognitive decline [27, 28]. However, the counterintuitive findings in our study suggest that higher grit was associated with worse subjective cognitive decline, and particularly higher among Black men. This supports a recent study's finding on grit and Black men's brain health [16]. A possible explanation for this finding is that individuals with high levels of grit may adopt unsustainably high‐effort trajectories, increasing their exposure to chronic stress and thereby amplifying worry and perceived cognitive decline [20]. Individuals with high grit may hold themselves to exceptionally high personal standards, potentially heightening their awareness of cognitive lapses [12]. This heightened self‐awareness may lead to increased self‐reported cognitive concerns, even in the absence of objective cognitive impairment.

It is worth noting, however, that such heightened self‐monitoring may not necessarily indicate true cognitive dysfunction. Rather, it may reflect adaptive vigilance — a heightened but functionally appropriate sensitivity to cognitive lapses that allows high‐grit individuals to detect subtle changes and self‐correct. This distinction has meaningful clinical implications: clinicians working with high‐grit individuals who report cognitive concerns should consider whether the presentation reflects genuine cognitive decline or an amplified yet non‐pathological awareness of normal cognitive variability. Neuropsychological testing or biomarker‐based evaluations (e.g., amyloid or tau assessment) could help differentiate hypervigilance‐driven self‐reports from clinically meaningful cognitive decline, serving as a critical checkpoint before attributing elevated SCD scores to true neurodegenerative risk in this population. Regarding the stronger effect of grit on SCD in Black men than women, Black men may face higher levels of stress and discrimination with implications for cognitive decline [29], and the cultural expectations on men to persevere and be self‐reliant in face of adversity may further contribute to heightened cognitive concerns [30].

Contrastingly, our study found that resilience is associated with better subjective cognitive outcomes, which aligns with previous research indicating that psychological resilience reduces stress‐related cognitive impairments [12, 31, 32]. The protective role of resilience in SCD underlines the importance of psychological coping mechanisms in cognitive health. Resilience has been linked to adaptive stress responses and emotional regulation, which may buffer against cognitive concerns [32]. This finding aligns with studies indicating that higher resilience in Black individuals is associated with lower depression and anxiety levels, both of which are known contributors to cognitive health issues [33]. At a biological level, resilience may confer protection against cognitive concerns through its influence on stress‐related neurobiological pathways. Specifically, resilience has been associated with more adaptive regulation of the hypothalamic‐pituitary‐adrenal (HPA) axis, resulting in attenuated cortisol reactivity and reduced allostatic load — a cumulative measure of physiological wear‐and‐tear that has been linked to accelerated cognitive aging [32]. Additionally, psychological resilience may promote neuroplasticity by supporting brain‐derived neurotrophic factor (BDNF) signaling and reducing neuroinflammatory processes implicated in synaptic degradation and cognitive decline. These neuroendocrine and neuroinflammatory pathways may, in part, explain the observed inverse association between resilience and SCD in the present study.

Interestingly, the association between higher resilience and positive cognitive outcomes was strongest in Black men compared to women [34]. This suggests that resilience may confer particular cognitive benefits for Black American men, whereas Black women's cognitive health may be influenced by other protective or risk factors beyond resilience [35]. These sex‐specific differences highlight the importance of considering intersectional influences on cognitive aging and suggest that resilience operates through distinct pathways across demographic groups. Future research should explore the mechanisms underlying these disparities, including the role of gendered stress exposure, coping resources, and sociocultural factors, to clarify how resilience contributes to cognitive health trajectories. Specifically, studies incorporating biomarker assessments of stress — such as cortisol reactivity, inflammatory markers (e.g., interleukin‐6, C‐reactive protein), and allostatic load indices — would help elucidate the biological pathways through which grit and resilience differentially affect cognitive health in Black men and women. Qualitative and mixed‐methods studies exploring how Black men and women perceive, report, and contextualize cognitive concerns would further enrich our understanding of the sociocultural factors driving the observed sex‐specific patterns. Additionally, examination of gendered differences in coping resource availability, social support networks, and structural stressor exposure would help disentangle the mechanisms underlying these effects.

### 4.1 Study Implications

The evidence of resilience as a protective factor against cognitive decline among Black Americans underscores its potential to mitigate or delay onset of cognitive decline in this group. Future studies may consider developing cognitive interventions that improves psychological resilience to prevent decline in cognitive functioning. Additionally, while strengthening community support programs may benefit Black women's cognitive health by enhancing resilience, interventions could also focus on improving Black men's health‐seeking behaviors to bolster their resilience against social stressors. Such interventions may include culturally tailored health and wellness initiatives (e.g., barbershop‐based health Interventions), accessible peer support networks (e.g., healing circles), and mentorship programs. However, to maximize protective effects, these efforts should be complemented by upstream, structural interventions, such as policies addressing systemic discrimination, economic inequities, and healthcare access, that can reinforce resilience at both the individual and community levels [36].

The findings of our study have broader implications for understanding disparities in cognitive decline, particularly among Black populations. The association between worse SCD and higher grit in Black Americans suggests that perseverance in the face of social adversity may be associated with greater cognitive concerns. This highlights the need to develop tailored and personalized interventions for people who report high levels of grit but experience elevated cognitive concerns. For high‐grit individuals, interventions should focus not on reducing goal‐directedness per se, but on fostering stress regulation and cognitive reappraisal skills that moderate the burden of sustained high‐effort striving. Specifically, mindfulness‐based stress reduction programs and cognitive‐behavioral approaches targeting the reframing of cognitive lapses may be particularly beneficial. These interventions can help individuals distinguish between normative everyday forgetting and clinically meaningful cognitive change. Healthcare providers should also be trained to screen for grit‐related hyperawareness of cognitive symptoms, particularly in Black male patients, and to integrate psychoeducation about the distinction between cognitive vigilance and cognitive decline into routine clinical encounters. For individuals with low resilience, interventions should prioritize resilience‐building components such as social support enhancement, positive reappraisal training, and community connectedness strategies. Further, interventions should be tailored to the unique effect of one's sex, as our study indicated that the association between grit and SCD was stronger in Black American men compared to women.

### 4.2 Limitations

Despite the contributions of the current research, we must acknowledge notable limitations. This study utilized a cross‐sectional design, which limits our ability to draw causal inferences. While we found significant associations between grit, resilience, and SCD, we cannot establish the directionality or causality of these relationships from the current design. A longitudinal study would offer greater understanding of how these associations unfold over time. Additionally, while the sample description as “middle‐to‐older aged” is consistent with the study's focus on midlife cognitive risk, it is worth noting that the age range spanned 27–79 years, with only 13.5% of the sample aged 55 or older and 5.7% aged 60 or above. Accordingly, findings may be most representative of middle‐aged Black adults, and future research should oversample older adults to more directly assess late‐life cognitive risk. SCD was measured using self‐reported assessments, which are inherently subjective and may not directly correspond to objective cognitive performance. Moreover, self‐reported SCD is susceptible to influence from other psychological and contextual factors, including depressive symptoms, perceived stress, neuroticism, and the salience of cognitive complaints, all of which may inflate or distort self‐perceptions of cognitive change independently of true cognitive status. Also, we controlled for several demographic factors. However, other unmeasured variables (e.g., chronic stress, socioeconomic adversity, health conditions (e.g., hypertension, diabetes), and access to healthcare) may have influenced the observed associations. Further, we collected the data online via CloudResearch, which may have introduced a potential for sampling bias. Participation may have been restricted to individuals with reliable internet access, adequate digital literacy, and willingness to engage in online studies, thereby limiting generalizability. Notably, characteristics required for successful participation in online research (e.g., digital literacy, technological self‐efficacy, and self‐motivated engagement)may themselves be positively correlated with grit and resilience, potentially resulting in an overrepresentation of higher‐grit and higher‐resilience individuals relative to the broader Black American community. This could attenuate the observed associations and warrants consideration when interpreting effect sizes. Despite these limitations, this study contributes to the growing body of literature on psychological factors and cognitive health by highlighting the potential role of grit and resilience in shaping subjective cognitive perceptions among Black American adults.

## 5 Conclusion

We examined the effect of grit and resilience on subjective cognitive decline in Black American adults. Our findings illuminate the intricate relationship between psychological coping strategies and cognitive health. Higher grit may be associated with greater subjective cognitive decline, while higher resilience is associated with decreased self‐report of cognitive decline. These findings highlight the importance of tailoring cognitive health interventions targeting psychological factors, sex, and racial/ethnic backgrounds. Future research should examine the role of psychosocial stressors, coping mechanisms, and neurobiological pathways in shaping subjective and objective cognitive outcomes. Longitudinal studies are particularly needed to determine whether baseline grit and resilience predict subsequent changes in SCD over time, and whether these associations persist or intensify as individuals advance into older age — findings that would establish directionality and inform targeted prevention efforts. Additionally, future studies should assess whether the observed associations replicate using objective cognitive measures, such as neuropsychological testing, performance‐based assessments, or neuroimaging biomarkers, to determine whether elevated self‐reported cognitive concerns in high‐grit individuals correspond to actual cognitive impairment or reflect adaptive vigilance.

## Author Contributions

SF, MC, OO, YJ, CN, and DKE conceptualized the study and contributed to study design. SF and OO conducted data analysis under the supervision of MC and DKE. YJ and CN provided methodological guidance and critical revisions. All authors contributed to drafting and reviewing the manuscript and approved the final version for submission.

## Funding

DE was supported by the National Institute on Aging (R00AG078286), the Alzheimer’s Association Research Fellowship (23AARFD‐1029261), and the Michigan Center for Contextual Factors in Alzheimer’s Disease (MCCFAD) enrichment grant. DE and CN were supported by NIMH/OBSSR grant to Michigan Integrative Wellbeing and Inequality (R25MH136652). YJ was supported by NCATS (TL1TR001997).

## Ethics Statement

Rutgers University IRB (#Pro2023000904). All participants provided informed consent.

## Conflicts of Interest

The authors declare no conflicts of interest.

## Sponsor's Role

The funding agencies had no role in the design, data collection, analysis, interpretation, or writing of the manuscript.

## Data Availability

Data are part of a larger project; details available from the first author. Standardized questionnaires are publicly accessible.
